# Glucocorticoid Metabolism in Obesity and Following Weight Loss

**DOI:** 10.3389/fendo.2020.00059

**Published:** 2020-02-20

**Authors:** Elina Akalestou, Laurent Genser, Guy A. Rutter

**Affiliations:** ^1^Section of Cell Biology and Functional Genomics, Division of Diabetes, Endocrinology and Metabolism, Department of Metabolism, Digestion and Reproduction, Imperial Centre for Translational and Experimental Medicine, Imperial College London, London, United Kingdom; ^2^Department of Digestive and Hepato-Pancreato-Biliary Surgery, Liver Transplantation, Assistance Publique-Hôpitaux de Paris, Pitié-Salpêtrière University Hospital, Institut Hospitalo-Universitaire ICAN, Sorbonne Université, Paris, France

**Keywords:** glucocorticoids, obesity, weight loss, bariatric surgery, 11β-HSD1

## Abstract

Glucocorticoids are steroid hormones produced by the adrenal cortex and are essential for the maintenance of various metabolic and homeostatic functions. Their function is regulated at the tissue level by 11β-hydroxysteroid dehydrogenases and they signal through the glucocorticoid receptor, a ligand-dependent transcription factor. Clinical observations have linked excess glucocorticoid levels with profound metabolic disturbances of intermediate metabolism resulting in abdominal obesity, insulin resistance and dyslipidaemia. In this review, we discuss the physiological mechanisms of glucocorticoid secretion, regulation and function, and survey the metabolic consequences of excess glucocorticoid action resulting from elevated release and activation or up-regulated signaling. Finally, we summarize the reported impact of weight loss by diet, exercise, or bariatric surgery on circulating and tissue-specific glucocorticoid levels and examine the therapeutic possibility of reversing glucocorticoid-associated metabolic disorders.

## Glucocorticoid Secretion

Glucocorticoid hormones are a class of corticosteroids, produced by the adrenal cortex primarily under the control of the hypothalamic-pituitary-adrenal (HPA) axis (1). Briefly, corticotropin-releasing hormone (CRH) and vasopressin are released from the hypothalamus and synergistically stimulate the secretion of stored adrenocorticotropic hormone (ACTH) from corticotrope cells in the anterior pituitary gland. Following this, ACTH is transported by the blood to the adrenal cortex, where it rapidly stimulates biosynthesis of corticosteroids such as cortisol from cholesterol (2). However, it recently became apparent (3) that the fine-tuning and regulation of the adrenal system is also controlled by ACTH-independent mechanisms. These include a temporal lag between stimulus-induced changes in circulating ACTH and in corticosteroid levels (4), adrenal corticosteroid metabolism and kinetics, and plasma protein binding (5–7). Moreover, dysregulation is observed in glucocorticoid secretion under pathological conditions (8, 9). An example of this is in obesity, where several studies in humans report (8, 10–12) an increase of cortisol secretion directly from the adrenal gland, yet the circulating plasma levels are normal, potentially due to higher metabolic clearance rate.

A variety of growth factors, neuropeptides, cytokines and adipokines have been demonstrated to affect adrenal secretions. Correspondingly, adrenocortical cells express receptors for each of these factors (13, 14). *In vitro* studies found that vital glucose regulating-peptides also have the capacity to regulate ACTH-stimulated glucocorticoid secretion. More specifically, insulin inhibits cortisol secretion from bovine adrenocortical cells (15), while glucagon inhibits cortisol secretion from human adrenocortical cells (16, 17). The satiety factor leptin was found to directly regulate adrenal secretions via its receptors on adrenocortical cells (18, 19). Likewise, the gut-derived incretin hormone glucagon-like peptide 1 (GLP-1) was also shown to inhibit glucocorticoid release from rat adrenal cortex in response to ACTH, by decreasing the activation of adenylate cyclase and by impairing the late steps of glucocorticoid synthesis (17). Nonetheless, intracerebroventricular, intravenous, and intraperitoneal administration of GLP-1 increased circulating levels of cortisol in rats, a change preceded by an increase in ACTH levels (20, 21). The mechanisms through which circulating GLP-1 activate the HPA axis remain to be elucidated, and may provide further evidence for the beneficial effect of GLP-1 receptor agonists in obesity and type 2 diabetes (T2DM) treatment.

### 11β-Hydroxysteroid Dehydrogenases

**Figure 1:**
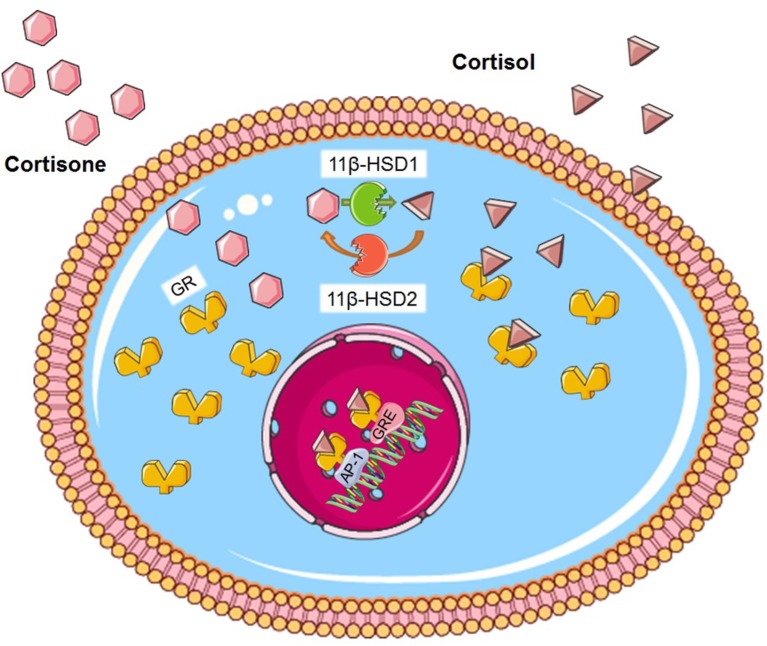
Glucocorticoid metabolism. Cortisone is activated to cortisol by the enzyme 11β-HSD1. Conversely, 11β-HSD2 inactivates cortisol by converting it into cortisone. GR is found in the cytosol. Cortisol binds on the GR and the ligand-receptor complex translocates to the nucleus where in can bind on GRE or to different transcription factors such as AP-1. Figure was created using Servier Medical Art.

Although the regulation of glucocorticoid secretion is an important means of their action, the effects of glucocorticoids on target tissues such as liver and adipose tissue are dependent on metabolism by 11β-hydroxysteroid dehydrogenases (11β-HSDs), with the notable exception of pancreatic β-cells (22). 11β-HSD1 is present in most cells and tissues and acts predominantly as an NADPH-dependent reductase to regenerate the active glucocorticoid receptor (GR) ligand cortisol (or corticosterone, in rodents) from inactive cortisone (Figure 1) (23). Conversely, 11β-HSD2 inactivates cortisol by converting it into cortisone, thereby protecting the mineralocorticoid receptor from cortisol ligands. 11β-HSD2 is largely expressed in the kidney, placenta, and colon whereas the principle sites of 11β-HSD1 expression are liver, adipose tissue and muscle (24). The A-ring reductases, 5-reductase types 1 and 2 (5R1 and 5R2) convert cortisol and cortisone to their dihydrometabolites, and these are next converted to tetrahydrometabolites through the action of 3-hydroxysteroid dehydrogenase (25, 26). Total glucocorticoid production can be estimated by analyzing the sum of glucocorticoid metabolites in a 24 h urinary sample. The relative excretion of cortisol to cortisonemetabolites [(5a-THF + THF + a-cortol)/(THE + a-cortolone)] reflects the global activity of 11β-HSD 1 (27).

Glucocorticoid metabolism at the tissue level is dysregulated in human obesity, with increased 5-reductase activity and decreased cortisol levels in the liver (28–30). Contrariwise, 11β-HSD1 activity is increased in adipose tissue, which increases tissue glucocorticoid levels. Mice overexpressing 11β-HSD1 in adipose tissue develop visceral obesity, insulin resistance, dyslipidaemia, and hypertension (31), while liver-specific 11β-HSD1 overexpression results in insulin resistance and hypertension, but not obesity (32). Interestingly, 11β-HSD1 appears to be absent from pancreatic α and β- cells though present in other cell types in the mouse and human islet (22, 33). Selective 11β-HSD1 inhibitors have been shown to lower glucose intolerance and reduce food intake and weight gain in hyperglycaemic mouse models (34–36). 11β-HSD1 knockout mice are resistant to hyperglycaemia when fed a high-fat diet and show reduced expression of mRNA encoding the key hepatic gluconeogenic enzyme phosphoenolpyruvate carboxykinase (PCK1) (37). Overall, these studies place 11β-HSD1 in a central position of cortisol metabolism and suggest that its inhibition may be a key target for diabetes and obesity treatments, especially as a mediator of insulin sensitivity. Nonetheless, the differential regulation of11β-HSD1 between organs implies a more complex pathway that may require equal attention to be paid to 11β-HSD2.

### Glucocorticoid Receptors (GR)

The function of glucocorticoids, both at a physiological and pharmacological level, is mediated by the GR. The GR is a member of the nuclear receptor superfamily of ligand-dependent transcription factors and its gene is regulated by both developmental and tissue-specific factors (38). Activated GR controls the expression of thousands of genes, either by inducing or inhibiting their transcription through DNA binding (39–41). The GR is composed of three main domains; the N-terminal transactivation, the central DNA-binding and the C-terminal ligand binding domains, with the central domain containing two zinc fingers. In the absence of glucocorticoids, GR is located primarily in the cytoplasm inside a large multi-protein complex. Once glucocorticoids are bound, GR dissociates from the complex and exposes two nuclear localization signals. GR then rapidly translocates into the nucleus through nuclear pores and, once inside, binds directly to glucocorticoid-responsive elements (GRE) and regulates the expression of target genes (Figure 1) (42). Our understanding of the role of the GR protein has changed dramatically within the last decade, as it is now accepted that the GR gene can be spliced into a large group of receptor isoforms, each with a different expression and function that widens the glucocorticoid diversity of GR action (43). Moreover, although this review focuses on the genomic mechanism of glucocorticoid function, several studies have demonstrated a rapid, non-genomic action mechanism that does not involve gene expression alteration and may involve an, as yet uncharacterised, receptor located at the plasma membrane (44–46).

Glucocorticoids acting through the GR regulate glucose metabolism in the liver, skeletal muscle, adipose tissue, and the pancreas, by controlling the expression of key enzymes. However, in obesity, cortisol levels remain at near normal concentrations, pointing to intracellular control of GR action in these circumstances. This suggests that GR polymorphisms might be responsible for the pathophysiology and evolution of obesity and diabetes (47, 48). The GR levels and activity have also been linked to diabetes pathogenesis, as increased hepatic GR mRNA induces activation of phosphoenolpyruvate carboxykinase (PEPCK), which results in hyperglycaemia and insulin resistance in diabetic obese mice and obese Zucker rats (49, 50). This observation was also made in human skeletal muscle, as increased GR was linked to the metabolic syndrome (51). Furthermore, general and hepatic inactivation of GR, achieved through the use of antagonists, was shown to improve glucose tolerance and insulin resistance in diabetic animals (52–54). The mass emerging observations on GR polymorphism could further indicate which receptor subtype can be a disease risk indicator, as well as improve specificity for GR antagonism treatment.

## Metabolic Function of Glucocorticoids

**Figure 2:**
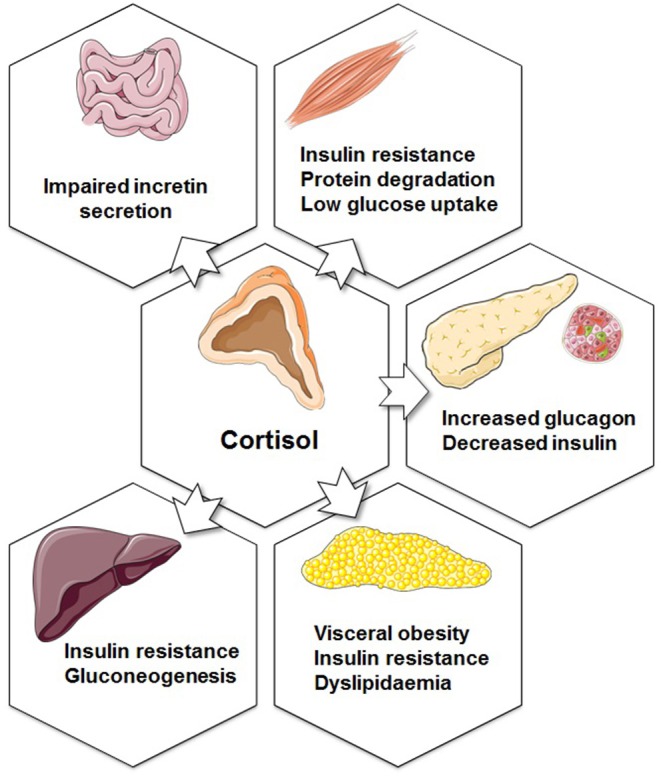
Metabolic functions of glucocorticoids. The effects of increased cortisol secretion on the endocrine pancreas, adipose tissue, liver, muscle, and gastrointestinal system. Figure was created using Servier Medical Art.

Glucocorticoids are involved in metabolic, inflammatory, cardiovascular, and behavioral processes. Thus, as mentioned above, they modulate the transcription of a variety of genes, including cytokines and chemokines, receptors, enzymes, adhesion molecules, and inhibitory proteins. Clinical observations linking high glucocorticoid levels to the metabolic syndrome provided evidence for their role on diabetes and obesity (55). The metabolic effects of glucocorticoids are linked to physiological mechanisms that are associated with hepatic and peripheral insulin resistance, hyperglycaemia, and dyslipidaemia. In the liver, glucocorticoids stimulate gluconeogenesis by activating PEPCK and glucose-6-phosphatase (G6Pase) (56). Moreover, in the fasting condition, glucocorticoids stimulate lipolysis in adipocytes, resulting in generation of glycerol to be utilized in gluconeogenesis and free fatty acids to be oxidized and get used as energy (57, 58). Although glucocorticoids are important for the maintenance of lipid homeostasis, excess glucocorticoids can result in an increase the circulating free fatty acids and induce lipid accumulation in skeletal muscle and liver, both of which are associated with insulin resistance (59–61). In rat skeletal muscle, glucocorticoid excess can also inhibit the translocation of GLUT4 glucose transporters to the plasma membrane in response to insulin, resulting in insulin resistance (Figure 2) (62). In human adipose tissue, glucocorticoids induce adipocyte differentiation leading to increased adiposity and insulin resistance (63–65).

The link between glucocorticoids and T2DM is strong, yet the effect of glucocorticoids on the endocrine pancreas remains controversial. The study of transgenic mice overexpressing the GR selectively in the β-cell indicated that glucocorticoids may directly inhibit insulin release *in vivo* (66). Correspondingly, several *in vitro* studies have demonstrated an inhibitory effect of glucocorticoids on mouse islets (67, 68). The function of 11β-HSD1 in mouse and human islets has been examined *in vitro*, and data suggest direct control over α-cell glucagon release and a paracrine effect on insulin secretion (69). In islets obtained from diabetic obese mice, glucocorticoid administration up regulated 11β-HSD1 causing impaired insulin secretion. This effect was abolished by GR antagonist or an 11β-HSD1 inhibitor (70).

Glucocorticoids have also been associated with pancreatic development. Treatment of fetal rats with dexamethasone reduced β-cell insulin content and examination of embryonic pancreata from glucocorticoid-treated animals revealed a decrease of insulin-producing cells and an increase in exocrine cells, possibly the result of down-regulation of pancreas maturation transcription factors such as Pdx1, Pax6, and Nkx6.1 (71, 72). However, islets from Wistar rats treated with dexamethasone for 5 days showed an enhanced insulin release in response to glucose, with an increased number of insulin granules docked at the plasma membrane observed in β-cells (73). Transgenic mice overexpressing 11β-HSD1 selectively to the β-cell displayed a reversal of high fat-induced β-cell failure. This was due to the expansion of β-cell population and the function of small islets, the latter being linked to protein kinase A and p21 signaling pathways (74). Finally, corticosterone, cortisol, and cortisone suppressed voltage-dependent Ca^2+^ channel activity in human and rodent β-cells, while in parallel amplifying cAMP signals and increasing the number of membrane-docked insulin secretory granules. Interestingly, no changes were observed in glucose-stimulated insulin secretion nor in the maximal ATP/ADP responses to glucose (75).

## Glucocorticoids and Weight Loss

### Diet and Exercise

Although a link between glucocorticoids and obesity has been reported in many studies, it is still unclear what changes following weight loss. Tomlinson et al. (76) reported the effects of weight loss on glucocorticoids in patients with obesity on very low calorie diet. Even though circulating cortisol and cortisone concentrations did not change with weight loss, the 0900h cortisol/cortisone ratio increased, indicating a shift in set-point toward cortisol generation consistent with increased 11β-HSD1 activity. Expression of 11β-HSD1 in whole adipose tissue did not change with weight loss but did increase in isolated adipocytes. No other corticosteroid was affected. A follow-up of this study (77) showed that total glucocorticoid secretion in this scenario decreased post-diet-induced weight loss. However, urinary steroid metabolite ratios that reflect 11β-HSD1 activity did not change. Moreover, increased cortisol availability within adipose tissue interstitial fluid was shown after weight loss.

In men with weight loss following 6 months of dieting, cortisol production rate, free cortisol levels and metabolic clearance rate did not change when compared to baseline (78). Nonetheless, with greater weight loss and decreased body fat, both cortisol production and free cortisol levels increased, whilst adipose 11β-HSD-1 decreased, compared to baseline (78). One week of caloric restriction in men also failed to reveal any change in cortisol levels (79). These results were further investigated in overweight/obese post-menopausal women following diet-induced weight loss with contradictory results. In one study (80), 11β-HSD1 expression in adipose tissue was decreased post-weight loss and this reduction was correlated with a reduction in BMI between baseline and 6 months post-diet initiation. In contrast, a previous study showed that weight reduction did not impact gene expression levels of 11β-HSD1 in adipose tissue (81).

Glucocorticoids respond acutely to changes in nutritional status, with cortisol levels increasing within minutes following a meal (82, 83). Given the discrepancies between studies, as well as differences in human and rodent data, it became clear that the type of meal in each diet had to be specified and specific dietary macronutrients were investigated with regards to their effect on metabolism (84). A high fat-low carbohydrate diet increased whole body 11β-HSD1 activity and decreased 5a- and 5b-reductase activities in men, compared with a moderate fat-moderate carbohydrate diet, with no effect on subcutaneous adipose tissue 11β-HSD1 (84). In rodents, although there was no difference between low carbohydrate and moderate carbohydrate diets, hepatic 11β-HSD1 mRNA was reduced in both diets when compared with a high fat Western diet (85).

Apart from diet, exercise is a common treatment for weight loss. However, exercise is also a form of metabolic stress which can stimulate the HPA axis and lead to increased levels of circulating glucocorticoids (86, 87). The importance of this has been investigated by separating acute intense and chronic voluntary exercises in rats on treadmill running, where intense exercise studies have shown higher corticosterone levels in urine, enlargement of the adrenal glands and reduced adrenal sensitivity to ACTH, as indicated by low ACTH-to-glucocorticoid ratio (88, 89), whether regular and voluntary exercise increased adrenal sensitivity, demonstrating that long-term training balances glucocorticoid fluctuations (86, 90).

Although increased glucocorticoid concentration can indeed have a negative impact in pancreatic β-cells alone (91), regular exercise can improve insulin sensitivity and glucose tolerance and this could be attributed to tissular, rather than systemic, glucocorticoid metabolism. It has previously been shown that exercise can lead to reduced expression of the glucocorticoid receptor and 11β-HSD1 in muscle and liver, yet unchanged circulating cortisol, in hamsters (87). Importantly, previous studies in humans and rodents have demonstrated that various types of exercise are able to improve insulin sensitivity, by recovering the function of specific insulin signaling proteins such as Akt and IRS-1 and also augment GLUT-4 expression (92–97). Insulin-stimulated glucose uptake in muscle, impaired by exposure to glucocorticoids, can also be enhanced by exercise. The effects of exercise appear to involve increased activation of insulin signal via Akt and IRS1 in rodents, as well as slow the glucocorticoid-induced muscle atrophy. This appears to improve through a combination of increases in mTOR and its downstream target p70S6K protein, and a small increase in MuRF-1 protein level, the latter a regulator of proteasome-dependent degradation of muscle proteins (94–96). To date, studies have been able to demonstrate that exercise is able to attenuate exogenous glucocorticoid-induced hyperglycaemia, while less is known about its effect on endogenous glucocorticoids.

Overall, reporting on the relationship between cortisol levels, obesity and weight loss has been challenging, especially if cortisol was measured in the serum, urine or saliva which would provide a snapshot in time rather than continuous reporting. This is most likely due to the fact that cortisol is secreted in a pulsatile manner, affected by the circadian rhythm, environmental circumstances and stress (98), which could even be diet-induced. In addition, gender and adiposity location also appear to have an effect on results. It is therefore important to remember that not all patients with obesity will demonstrate similar cortisol secretion and metabolism, especially at baseline level. Chronic cortisol measurement may provide a more appropriate and ubiquitous way to report changes in glucocorticoid metabolism, potentially by using hair cortisol measurements (99–102), which has been shown to be a novel and accurate way to measure average systemic cortisol levels.

### Bariatric Surgery

Bariatric surgery, and particularly Roux-en-Y gastric bypass (RYGB), causes sustained weight loss in individuals with morbid obesity, as well as dramatic and rapid improvement of T2DM, dyslipidaemia, hypertension and a significant reduction of cardiovascular disease and death (103). Although the effects of RYGB on diabetes were initially attributed to the substantial weight loss subsequent to post-operative restriction of nutrient intake and/or absorption, the same effect was demonstrated in rodents that did not experience weight loss, suggesting a mechanism of action that is weight independent. Moreover, the improvements are observed within hours and days, long before substantial weight loss occurred.

In an effort to understand the underlying mechanisms of diabetes remission, this observation has since led to numerous investigations on the glucoregulatory role of the gastrointestinal tract, including the role of an increase in observed post-operative GLP-1 concentration. This includes the “hindgut hypothesis” which holds that euglycaemic effects are derived from the expedited delivery of nutrients to the distal intestine, where GLP-1 is primarily secreted, and therefore enhances the insulin signal that improves glucose metabolism (104). Few data are available regarding the specific modifications in glucocorticoid metabolism after bariatric surgery in human obesity. Given the numerous endocrine changes following surgery, one might expect to have a conclusive picture of glucocorticoid metabolism post-operatively. The fact that this is the case due to different surgical procedures, as well as alterations of the same procedures, which can cause perioperative stress and different levels of caloric restriction and weight loss.

Several observations have linked bariatric surgery to glucocorticoid metabolism, notably in the context of post-operative adipose tissue reduction. Woods et al. investigated the activity and expression of 11β-HSD1 in hepatic and adipose tissue before and ~14 months after RYGB, revealing a post-operative increase in hepatic 11β-HSD1 activity, as inferred from a by raised serum cortisol/costisone ratio (F/E) (105). However, subcutaneous adipose tissue 11β-HSD1 activity was decreased, as showed by the tissue's F/E. Moreover, total urinary cortisol metabolites were reduced, suggesting a reduction in HPA axis activity (105). The decrease of subcutaneous adipose tissue 11β-HSD1 activity at 1 and 2 years after gastric bypass surgery in humans has also been reported through both mRNA expression and urine and adipose tissue F/E, along with positive changes in insulin sensitivity, circulating leptin, and adiponectin, and peripheral glucocorticoid metabolism (106–108). Additionally, intra-adipose levels of cortisone, rather than cortisol, demonstrated the most obvious changes, suggesting that the altered glucocorticoid metabolism after weight loss may be an adaptive response to insufficient levels of adipose cortisol. Of note, supplementary data in Methlie et al. (108), show that obese patients at 1 year post-RYGB demonstrated a significant reduction of adipose tissue F/E, when compared to non-obese controls. This observation points to an effect potentially caused by more than surgery-induced weight loss and may include the gastrointestinal tract manipulation itself. One potential mechanism could be the effect of post-operatively increased GLP-1 on glucocorticoid regulation, as discussed before. Taken together, the reduction in glucocorticoid exposure in subjects with obesity may represent an additional possible contribution to the health benefits of bariatric surgery.

## Discussion

Glucocorticoids are steroid hormones that are crucial for the preservation of homeostasis. Their physiological and therapeutic effects have made them key targets for drug development, primarily as anti-inflammatory agents. Nonetheless, their side effects can be severe, especially in terms of metabolism. In this review, we provide an overview of the physiology glucocorticoid regulation, metabolism, and signaling pathways, as well as their effects on obesity and diabetes. We also discussed the ability of weight loss to reverse some of these effects.

Overall, high glucocorticoid levels are associated with hyperglycaemia and insulin resistance and affect key metabolic organs such as the pancreas, liver, muscle and adipose tissue (28, 30, 107, 109). Of note, the exact mechanism of glucocorticoid action remains controversial in pancreatic islets, especially in insulin secretion during glucocorticoid treatment (66–68). Glucocorticoid metabolism is largely controlled by 11β-HSD1 and GR and has been shown to be strongly linked to Body Mass Index, as intra-adipose cortisol levels are increased relative to inactive cortisone in the obese state, even if circulating cortisol levels remain stable. Weight loss through diet, exercise, and bariatric surgery is linked to reduced glucocorticoid secretion and function, possibly through the recovery of insulin sensitivity and function of insulin sensitivity proteins. In all three weight loss interventions, the expression levels of 11β-HSD1 in adipose tissue and liver remains a matter of debate, as studies report both increasing and decreasing levels (78–80, 107, 108). Moreover, acute cortisol measurements are presented, through serum and plasma samples. It is therefore vital to measure chronic cortisol levels to get a longer-term picture of cortisol alterations, and investigate the role of 11β-HSD1 and GR following rapid weight loss in patients, especially following bariatric surgery which is currently the least published field. This will help us determine if the positive observations on glucocorticoid metabolism are due to weight loss or if direct or indirect gastrointestinal manipulation can also have an effect.

## Author Contributions

EA prepared the first draft and revised the manuscript. LG and GR revised the manuscript.

### Conflict of Interest

GR has received grant funds from Servier Laboratories and Sun Pharmaceutical Industries Ltd. These funders were not involved in any of the studies discussed here. The remaining authors declare that the research was conducted in the absence of any commercial or financial relationships that could be construed as a potential conflict of interest.

## References

[1] Smith SM Vale WW. The role of the hypothalamic-pituitary-adrenal axis in neuroendocrine responses to stress. Dialogues Clin Neurosci. (2006) 8:383–95. 17290797 10.31887/DCNS.2006.8.4/ssmith PMC3181830

[2] Adriana Del Rey GC Besedovsky H The Hypothalamus-Pituitary-Adrenal Axis. The Hypothalamus-Pituitary-Adrenal Axis. 7.1 Edn. Amsterdam: Elsevier Science (2008).

[3] Bornstein SR Engeland WC Ehrhart-Bornstein M Herman JP. Dissociation of ACTH and glucocorticoids. Trends Endocrinol Metab. (2008) 19:175–80. 10.1016/j.tem.2008.01.009 18394919

[4] Engeland WC Shinsako J Winget CM Vernikos-Danellis J Dallman MF. Circadian patterns of stress-induced ACTH secretion are modified by corticosterone responses. Endocrinology. (1977) 100:138–47. 10.1210/endo-100-1-138 187404

[5] Vazquez DM Morano MI Taylor L Akil H. Kinetics of radiolabeled adrenocorticotropin hormone in infant and weanling rats. J Neuroendocrinol. (1997) 9:529–36. 10.1046/j.1365-2826.1997.00608.x 15305571

[6] Dhillo WS Kong WM Le Roux CW Alaghband-Zadeh J Jones J Carter G. Cortisol-binding globulin is important in the interpretation of dynamic tests of the hypothalamic–pituitary–adrenal axis. Eur J Endocrinol. (2002) 146:231–5. 10.1530/eje.0.1460231 11834433

[7] Gardner DS Fletcher AJ Fowden AL Giussani DA. Plasma adrenocorticotropin and cortisol concentrations during acute hypoxemia after a reversible period of adverse intrauterine conditions in the ovine fetus during late gestation. Endocrinology. (2001) 142:589–98. 10.1210/endo.142.2.7980 11159829

[8] Vierhapper H Nowotny P Waldhausl W. Production rates of cortisol in obesity. Obes Res. (2004) 12:1421–5. 10.1038/oby.2004.178 15483206

[9] Stewart PM Boulton A Kumar S Clark PM Shackleton CH. Cortisol metabolism in human obesity: impaired cortisone–>cortisol conversion in subjects with central adiposity. J Clin Endocrinol Metab. (1999) 84:1022–7. 10.1210/jc.84.3.1022 10084590

[10] Andrew R Phillips DI Walker BR. Obesity and gender influence cortisol secretion and metabolism in man. J Clin Endocrinol Metab. (1998) 83:1806–9. 10.1210/jcem.83.5.4951 9589697

[11] Phillips DIW Barker DJP Fall CHD Seckl JR Whorwood CB Wood PJ. Elevated plasma cortisol concentrations: a link between low birth weight and the insulin resistance syndrome? J Clin Endocr Metab. (1998) 83:757–60. 10.1210/jcem.83.3.4634 9506721

[12] Strain GW Zumoff B Kream J Strain JJ Levin J Fukushima D. Sex Difference in the influence of obesity on the 24-hr mean plasma-concentration of cortisol. Metabolism. (1982) 31:209–12. 10.1016/0026-0495(82)90054-3 7078409

[13] Bornstein SR Chrousos GP. Clinical review 104: Adrenocorticotropin (ACTH)- and non-ACTH-mediated regulation of the adrenal cortex: neural and immune inputs. J Clin Endocrinol Metab. (1999) 84:1729–36. 10.1210/jcem.84.5.5631 10323408

[14] Lamounier-Zepter V Ehrhart-Bornstein M Bornstein SR. Metabolic syndrome and the endocrine stress system. Horm Metab Res. (2006) 38:437–41. 10.1055/s-2006-947837 16933178

[15] Klein NA Andersen RN Casson PR Buster JE Kramer RE. Mechanisms of insulin-inhibition of acth-stimulated steroid-secretion by cultured bovine adrenocortical-cells. J Steroid Biochem. (1992) 41:11–20. 10.1016/0960-0760(92)90219-9 1370906

[16] Andreis PG Malendowicz LK Neri G Tortorella C Nussdorfer GG. Effects of glucagon and glucagon-like peptide-1 on glucocorticoid secretion of dispersed rat adrenocortical cells. Life Sci. (1999) 64:2187–97. 10.1016/S0024-3205(99)00170-8 10374908

[17] Mazzocchi G Gottardo L Aragona F Albertin G Nussdorfer GG. Glucagon inhibits ACTH-stimulated cortisol secretion from dispersed human adrenocortical cells by activating unidentified receptors negatively coupled with the adenylate cyclase cascade. Horm Metab Res. (2000) 32:265–8. 10.1055/s-2007-978633 10965931

[18] Bornstein SR Uhlmann K Haidan A EhrhartBornstein M Scherbaum WA. Evidence for a novel peripheral action of leptin as a metabolic signal to the adrenal gland - Leptin inhibits cortisol release directly. Diabetes. (1997) 46:1235–8. 10.2337/diab.46.7.1235 9200662

[19] Glasow A Bornstein SR. Leptin and the adrenal gland. Eur J Clin Invest. (2000) 30:39–45. 10.1046/j.1365-2362.2000.0300s3039.x 11281366

[20] Kinzig KP D'Alessio DA Herman JP Sakai RR Vahl TP Figueredo HF. CNS glucagon-like peptide-1 receptors mediate endocrine and anxiety responses to interoceptive and psychogenic stressors. J Neurosci. (2003) 23:6163–70. 10.1523/JNEUROSCI.23-15-06163.2003 12867498 PMC6740553

[21] Gil-Lozano M Perez-Tilve D Alvarez-Crespo M Martis A Fernandez AM Catalina PAF. GLP-1(7-36)-amide and exendin-4 stimulate the HPA axis in rodents and humans. Endocrinology. (2010) 151:2629–40. 10.1210/en.2009-0915 20363879

[22] Pullen TJ Huising MO Rutter GA. Analysis of purified pancreatic islet beta and alpha cell transcriptomes reveals 11beta-hydroxysteroid dehydrogenase (Hsd11b1) as a novel disallowed gene. Front Genet. (2017) 8:41. 10.3389/fgene.2017.00041 28443133 PMC5385341

[23] Rebuffescrive M Bronnegard M Nilsson A Eldh J Gustafsson JA Bjorntorp P Steroid-hormone receptors in human adipose tissues. J Clin Endocr Metab. (1990) 71:1215–9. 10.1210/jcem-71-5-1215 2229280

[24] Liu YJ Nakagawa Y Ohzeki T Gene expression of 11 beta-hydroxysteroid dehydrogenase type 1 and type 2 in the kidneys of insulin-dependent diabetic rats. Hypertension. (1998) 31:885–9. 10.1161/01.HYP.31.3.885 9495277

[25] Palermo M Shackleton CHL Mantero F Stewart PM. Urinary free cortisone and the assessment of 11 beta-hydroxysteroid dehydrogenase activity in man. Clin Endocrinol. (1996) 45:605–11. 10.1046/j.1365-2265.1996.00853.x 8977758

[26] Rogers SL Hughes BA Jones CA Freedman L Smart K Taylor N Diminished 11 beta-hydroxysteroid dehydrogenase type 2 activity is associated with decreased weight and weight gain across the first year of life. J Clin Endocr Metab. (2014) 99:E821–E31. 10.1210/jc.2013-3254 24517145

[27] Andrew R Smith K Jones GC Walker BR Distinguishing the activities of 11 beta-hydroxysteroid dehydrogenases *in vivo* using isotopically labeled cortisol. J Clin Endocr Metab. (2002) 87:277–85. 10.1210/jc.87.1.277 11788660

[28] Livingstone DE Jones GC Smith K Jamieson PM Andrew R Kenyon CJ. Understanding the role of glucocorticoids in obesity: tissue-specific alterations of corticosterone metabolism in obese Zucker rats. Endocrinology. (2000) 141:560–3. 10.1210/endo.141.2.7297 10650936

[29] Rask E Olsson T Soderberg S Andrew R Livingstone DE Johnson O. Tissue-specific dysregulation of cortisol metabolism in human obesity. J Clin Endocrinol Metab. (2001) 86:1418–21. 10.1210/jcem.86.3.7453 11238541

[30] Stomby A Andrew R Walker BR Olsson T. Tissue-specific dysregulation of cortisol regeneration by 11betaHSD1 in obesity: has it promised too much? Diabetologia. (2014) 57:1100–10. 10.1007/s00125-014-3228-6 24710966

[31] Masuzaki H Paterson J Shinyama H Morton NM Mullins JJ Seckl JR. A transgenic model of visceral obesity and the metabolic syndrome. Science. (2001) 294:2166–70. 10.1126/science.1066285 11739957

[32] Paterson JM Morton NM Fievet C Kenyon CJ Holmes MC Staels B Metabolic syndrome without obesity: hepatic overexpression of 11 beta-hydroxysteroid dehydrogenase type 1 in transgenic mice. Proc Natl Acad Sci USA. (2004) 101:7088–93. 10.1073/pnas.0305524101 15118095 PMC406470

[33] Fine NHF Doig CL Elhassan YS Vierra NC Marchetti P Bugliani M. Glucocorticoids reprogram beta-cell signaling to preserve insulin secretion. Diabetes. (2018) 67:278–90. 10.2337/db16-1356 29203512 PMC5780059

[34] Alberts P Nilsson C Selen G Engblom LOM Edling NHM Norling S. Selective inhibition of 11 beta-hydroxysteroid dehydrogenase type 1 improves hepatic insulin sensitivity in hyperglycemic mice strains. Endocrinology. (2003) 144:4755–62. 10.1210/en.2003-0344 12960099

[35] Wang SJY Birtles S de Schoolmeester J Swales J Moody G Hislop D Inhibition of 11 beta-hydroxysteroid dehydrogenase type 1 reduces food intake and weight gain but maintains energy expenditure in diet-induced obese mice. Diabetologia. (2006) 49:1333–7. 10.1007/s00125-006-0239-y 16612591

[36] Liu YJ Nakagawa Y Wang Y Liu LM Du HW Wang W. Reduction of hepatic glucocorticoid receptor and hexose-6-phosphate dehydrogenase expression ameliorates diet-induced obesity and insulin resistance in mice. J Mol Endocrinol. (2008) 41:53–64. 10.1677/JME-08-0004 18524870 PMC2954685

[37] Kotelevtsev Y Holmes MC Burchell A Houston PM Schmoll D Jamieson P 11 beta-hydroxysteroid dehydrogenase type 1 knockout mice show attenuated glucocorticoid-inducible responses and resist hyperglycemia on obesity or stress. Proc Natl Acad Sci USA. (1997) 94:14924–9. 10.1073/pnas.94.26.14924 9405715 PMC25139

[38] Kalinyak JE Griffin CA Hamilton RW Bradshaw JG Perlman AJ Hoffman AR. Developmental and hormonal-regulation of glucocorticoid receptor messenger-RNA in the rat. J Clin Invest. (1989) 84:1843–8. 10.1172/JCI114370 2592562 PMC304063

[39] Evans RM. The steroid and thyroid-hormone receptor superfamily. Science. (1988) 240:889–95. 10.1126/science.3283939 3283939 PMC6159881

[40] Oakley RH Cidlowski JA. The biology of the glucocorticoid receptor: new signaling mechanisms in health and disease. J Allergy Clin Immunol. (2013) 132:1033–44. 10.1016/j.jaci.2013.09.007 24084075 PMC4084612

[41] Scheschowitsch K Leite JA Assreuy J. New insights in glucocorticoid receptor signaling-more than just a ligand-binding receptor. Front Endocrinol. (2017) 8:16. 10.3389/fendo.2017.00016 28220107 PMC5292432

[42] Beato M. Gene-Regulation by Steroid-Hormones. Cell. (1989) 56:335–44. 10.1016/0092-8674(89)90237-7 2644044

[43] van Rossum EFC Lamberts SWJ. Polymorphisms in the glucocorticoid receptor gene and their associations with metabolic parameters and body composition. Recent Prog Horm Res. (2004) 59:333–57. 10.1210/rp.59.1.333 14749509

[44] Johnstone WM 3rd Honeycutt JL Deck CA Borski RJ. Nongenomic glucocorticoid effects and their mechanisms of action in vertebrates. Int Rev Cell Mol Biol. (2019) 346:51–96. 10.1016/bs.ircmb.2019.03.004 31122395

[45] Borski RJ. Nongenomic membrane actions of glucocorticoids in vertebrates. Trends Endocrinol Metab. (2000) 11:427–36. 10.1016/S1043-2760(00)00325-8 11091121

[46] Song IH Buttgereit F. Non-genomic glucocorticoid effects to provide the basis for new drug developments. Mol Cell Endocrinol. (2006) 246:142–6. 10.1016/j.mce.2005.11.012 16388891

[47] Moraitis AG Block T Nguyen D Belanoff JK. The role of glucocorticoid receptors in metabolic syndrome and psychiatric illness. J Steroid Biochem. (2017) 165:114–20. 10.1016/j.jsbmb.2016.03.023 27002803

[48] Majer-Lobodzinska A Adamiec-Mroczek J. Glucocorticoid receptor polymorphism in obesity and glucose homeostasis. Adv Clin Exp Med. (2017) 26:143–8. 10.17219/acem/41231 28397446

[49] Liu YJ Nakagawa Y Wang Y Sakurai R Tripathi PV Lutfy K. Increased glucocorticoid receptor and 11 beta-hydroxysteroid dehydrogenase type 1 expression in hepatocytes may contribute to the phenotype of type 2 diabetes in db/db mice. Diabetes. (2005) 54:32–40. 10.2337/diabetes.54.1.32 15616008

[50] Jenson M Kilroy G York DA Braymer D. Abnormal regulation of hepatic glucocorticoid receptor mRNA and receptor protein distribution in the obese Zucker rat. Obes Res. (1996) 4:133–43. 10.1002/j.1550-8528.1996.tb00525.x 8681046

[51] Whorwood CB Donovan SJ Flanagan D Phillips DIW Byrne CD. Increased glucocorticoid receptor expression in human skeletal muscle cells may contribute to the pathogenesis of the metabolic syndrome. Diabetes. (2002) 51:1066–75. 10.2337/diabetes.51.4.1066 11916927

[52] Watts LM Manchem VP Leedom TA Rivard AL McKay RA Bao DJ. Reduction of hepatic and adipose tissue glucocorticoid receptor expression with antisense oligonucleotides improves hyperglycemia and hyperlipidemia in diabetic rodents without causing systemic glucocorticoid antagonism. Diabetes. (2005) 54:1846–53. 10.2337/diabetes.54.6.1846 15919808

[53] Opherk C Tronche F Kellendonk C Kohlmuller D Schulze A Schmid W. Inactivation of the glucocorticoid receptor in hepatocytes leads to fasting hypoglycemia and ameliorates hyperglycemia in streptozotocin-induced diabetes mellitus. Mol Endocrinol. (2004) 18:1346–53. 10.1210/me.2003-0283 15031319

[54] John K Marino JS Sanchez ER Hinds TD Jr The glucocorticoid receptor: cause of or cure for obesity? Am J Physiol Endocrinol Metab. (2016) 310:E249–57. 10.1152/ajpendo.00478.2015 26714851 PMC4838130

[55] Andrew R Gale CR Walker BR Seckl JR Martyn CN. Glucocorticoid metabolism and the metabolic syndrome: associations in an elderly cohort. Exp Clin Endocr Diab. (2002) 110:284–90. 10.1055/s-2002-34591 12373632

[56] Argaud D Zhang Q Pan WS Maitra S Pilkis SJ Lange AJ. Regulation of rat liver glucose-6-phosphatase gene expression in different nutritional and hormonal states—Gene structure and 5'-flanking sequence. Diabetes. (1996) 45:1563–71. 10.2337/diab.45.11.1563 8866562

[57] Berdanier CD. Role of glucocorticoids in the regulation of lipogenesis. FASEB J. (1989) 3:2179–83. 10.1096/fasebj.3.10.2666232 2666232

[58] Xu C He J Jiang H Zu L Zhai W Pu S. Direct effect of glucocorticoids on lipolysis in adipocytes. Mol Endocrinol. (2009) 23:1161–70. 10.1210/me.2008-0464 19443609 PMC5419195

[59] Samuel VT Petersen KF Shulman GI. Lipid-induced insulin resistance: unravelling the mechanism. Lancet. (2010) 375:2267–77. 10.1016/S0140-6736(10)60408-4 20609972 PMC2995547

[60] Petersen KF Shulman GI. Etiology of insulin resistance. Am J Med. (2006) 119(5 Suppl 1):S10–6. 10.1016/j.amjmed.2006.01.009 16563942 PMC2995525

[61] Dourakis SP Sevastianos VA Kaliopi P. Acute severe steatohepatitis related to prednisolone therapy. Am J Gastroenterol. (2002) 97:1074–5. 10.1111/j.1572-0241.2002.05644.x 12003403

[62] Dimitriadis G Leighton B ParryBillings M Sasson S Young M Krause U. Effects of glucocorticoid excess on the sensitivity of glucose transport and metabolism to insulin in rat skeletal muscle. Biochem J. (1997) 321:707–12. 10.1042/bj3210707 9032457 PMC1218126

[63] Hauner H Entenmann G Wabitsch M Gaillard D Ailhaud G Negrel R. Promoting effect of glucocorticoids on the differentiation of human adipocyte precursor cells cultured in a chemically defined medium. J Clin Invest. (1989) 84:1663–70. 10.1172/JCI114345 2681273 PMC304034

[64] Hauner H Schmid P Pfeiffer EF. Glucocorticoids and insulin promote the differentiation of human adipocyte precursor cells into fat-cells. J Clin Endocr Metab. (1987) 64:832–5. 10.1210/jcem-64-4-832 3546356

[65] Gathercole LL Morgan SA Bujalska IJ Hauton D Stewart PM Tomlinson JW. Regulation of lipogenesis by glucocorticoids and insulin in human adipose tissue. PLoS ONE. (2011) 6:e26223. 10.1371/journal.pone.0026223 22022575 PMC3194822

[66] Delaunay F Khan A Cintra A Davani B Ling ZC Andersson A. Pancreatic beta cells are important targets for the diabetogenic effects of glucocorticoids. J Clin Invest. (1997) 100:2094–8. 10.1172/JCI119743 9329975 PMC508401

[67] Gremlich S Roduit R Thorens B. Dexamethasone induces posttranslational degradation of GLUT2 and inhibition of insulin secretion in isolated pancreatic beta cells - Comparison with the effects of fatty acids. J Biol Chem. (1997) 272:3216–22. 10.1074/jbc.272.6.3216 9013557

[68] Lambillotte C Gilon P Henquin JC. Direct glucocorticoid inhibition of insulin secretion - An *in vitro* study of dexamethasone effects in mouse islets. J Clin Invest. (1997) 99:414–23. 10.1172/JCI119175 9022074 PMC507814

[69] Swali A Walker EA Lavery GG Tomlinson JW Stewart PM 11 beta-hydroxysteroid dehydrogenase type 1 regulates insulin and glucagon secretion in pancreatic islets. Diabetologia. (2008) 51:2003–11. 10.1007/s00125-008-1137-2 18779947

[70] Ortsater H Alberts P Warpman U Engblom LOM Abrahmsen L Bergsten P Regulation of 11 beta-hydroxysteroid dehydrogenase type 1 and glucose-stimulated insulin secretion in pancreatic islets of Langerhans. Diabetes-Metab Res. (2005) 21:359–66. 10.1002/dmrr.525 15586384

[71] Gesina E Tronche F Herrera P Duchene B Tales W Czernichow P. Dissecting the role of glucocorticoids on pancreas development. Diabetes. (2004) 53:2322–9. 10.2337/diabetes.53.9.2322 15331541

[72] Shen CN Seckl JR Slack JMW Tosh D. Glucocorticoids suppress beta-cell development and induce hepatic metaplasia in embryonic pancreas. Biochem J. (2003) 375:41–50. 10.1042/bj20030140 14509268 PMC1223676

[73] Rafacho A Marroqui L Taboga SR Abrantes JLF Silveira LR Boschero AC. Glucocorticoids *in vivo* induce both insulin hypersecretion and enhanced glucose sensitivity of stimulus-secretion coupling in isolated rat islets. Endocrinology. (2010) 151:85–95. 10.1210/en.2009-0704 19880808

[74] Turban S Liu XX Ramage L Webster SP Walker BR Dunbar DR Optimal elevation of beta-cell 11 beta-hydroxysteroid dehydrogenase type 1 is a compensatory mechanism that prevents high-fat diet induced beta-cell failure. Diabetes. (2012) 61:642–52. 10.2337/db11-1054 22315313 PMC3282808

[75] Fine NHF Doig CL Elhassan Y Marchetti P Bugliani M Piemonti L Glucocorticoids re-programme the beta cell signalling cassette to preserve functional identity and insulin secretion. Diabetologia. (2017) 60:S83.

[76] Tomlinson JW Moore JS Clark PM Holder G Shakespeare L Stewart PM. Weight loss increases 11beta-hydroxysteroid dehydrogenase type 1 expression in human adipose tissue. J Clin Endocrinol Metab. (2004) 89:2711–6. 10.1210/jc.2003-031376 15181046 PMC7611657

[77] Tomlinson JW Finney J Hughes BA Hughes SV Stewart PM Reduced glucocorticoid production rate, decreased 5 alpha-reductase activity, and adipose tissue insulin sensitization after weight loss. Diabetes. (2008) 57:1536–43. 10.2337/db08-0094 18340018 PMC7611651

[78] Purnell JQ Kahn SE Samuels MH Brandon D Loriaux DL Brunzell JD. Enhanced cortisol production rates, free cortisol, and 11beta-HSD-1 expression correlate with visceral fat and insulin resistance in men: effect of weight loss. Am J Physiol Endocrinol Metab. (2009) 296:E351–7. 10.1152/ajpendo.90769.2008 19050176 PMC2645022

[79] Johnstone AM Faber P Andrew R Gibney ER Elia M Lobley G. Influence of short-term dietary weight loss on cortisol secretion and metabolism in obese men. Eur J Endocrinol. (2004) 150:185–94. 10.1530/eje.0.1500185 14763916

[80] Stomby A Simonyte K Mellberg C Ryberg M Stimson RH Larsson C. Diet-induced weight loss has chronic tissue-specific effects on glucocorticoid metabolism in overweight postmenopausal women. Int J Obes. (2015) 39:814–9. 10.1038/ijo.2014.188 25349058

[81] Engeli S Bohnke J Feldpausch M Gorzelniak K Heintze U Janke J. Regulation of 11beta-HSD genes in human adipose tissue: influence of central obesity and weight loss. Obes Res. (2004) 12:9–17. 10.1038/oby.2004.3 14742837

[82] Basu R Singh R Basu A Johnson CM Rizza RA. Effect of nutrient ingestion on total-body and splanchnic cortisol production in humans. Diabetes. (2006) 55:667–74. 10.2337/diabetes.55.03.06.db05-1335 16505229

[83] Wake DJ Homer NZ Andrew R Walker BR. Acute *in vivo* regulation of 11beta-hydroxysteroid dehydrogenase type 1 activity by insulin and intralipid infusions in humans. J Clin Endocrinol Metab. (2006) 91:4682–8. 10.1210/jc.2006-0819 16954164

[84] Stimson RH Johnstone AM Homer NZ Wake DJ Morton NM Andrew R. Dietary macronutrient content alters cortisol metabolism independently of body weight changes in obese men. J Clin Endocrinol Metab. (2007) 92:4480–4. 10.1210/jc.2007-0692 17785367

[85] Stimson RH Lobley GE Maraki I Morton NM Andrew R Walker BR. Effects of proportions of dietary macronutrients on glucocorticoid metabolism in diet-induced obesity in rats. PLoS ONE. (2010) 5:e8779. 10.1371/journal.pone.0008779 20098742 PMC2808251

[86] Campbell JE Rakhshani N Fediuc S Bruni S Riddell MC. Voluntary wheel running initially increases adrenal sensitivity to adrenocorticotrophic hormone, which is attenuated with long-term training. J Appl Physiol. (1985). (2009) 106:66–72. 10.1152/japplphysiol.91128.2008 19008482

[87] Coutinho AE Campbell JE Fediuc S Riddell MC Effect of voluntary exercise on peripheral tissue glucocorticoid receptor content and the expression and activity of 11 beta-HSD1 in the Syrian hamster. J Appl Physiol. (2006) 100:1483–8. 10.1152/japplphysiol.01236.2005 16357069

[88] Chennaoui M Gomez Merino D Lesage J Drogou C Guezennec CY. Effects of moderate and intensive training on the hypothalamo-pituitary-adrenal axis in rats. Acta Physiol Scand. (2002) 175:113–21. 10.1046/j.1365-201X.2002.00971.x 12028131

[89] Moraska A Deak T Spencer RL Roth D Fleshner M. Treadmill running produces both positive and negative physiological adaptations in Sprague-Dawley rats. Am J Physiol-Reg I. (2000) 279:R1321–R9. 10.1152/ajpregu.2000.279.4.R1321 11004000

[90] Dishman RK Bunnell BN Youngstedt SD Yoo HS Mougey EH Meyerhoff JL. Activity wheel running blunts increased plasma adrenocorticotrophin (ACTH) after footshock and cage-switch stress. Physiol Behav. (1998) 63:911–7. 10.1016/S0031-9384(98)00017-1 9618016

[91] Fichna M Fichna P. Glucocorticoids and beta-cell function. Endokrynol Pol. (2017) 68:568–73. 10.5603/EP.2017.0060 29168546

[92] Tanner CJ Koves TR Cortright RL Pories WJ Kim YB Kahn BB. Effect of short-term exercise training on insulin-stimulated PI 3-kinase activity in middle-aged men. Am J Physiol Endocrinol Metab. (2002) 282:E147–53. 10.1152/ajpendo.2002.282.1.E147 11739095

[93] Luciano E Carneiro EM Carvalho CRO Carvalheira JBC Peres SB Reis MAB. Endurance training improves responsiveness to insulin and modulates insulin signal transduction through the phosphatidylinositol 3-kinase/Akt-1 pathway. Eur J Endocrinol. (2002) 147:149–57. 10.1530/eje.0.1470149 12088932

[94] Krug ALO Macedo AG Zago AS Rush JWE Santos CF Amaral SL. High-intensity resistance training attenuates dexamethasone-induced muscle atrophy. Muscle Nerve. (2016) 53:779–88. 10.1002/mus.24906 26355638

[95] Barel M Perez OAB Giozzet VA Rafacho A Bosqueiro JR do Amaral SL. Exercise training prevents hyperinsulinemia, muscular glycogen loss and muscle atrophy induced by dexamethasone treatment. Eur J Appl Physiol. (2010) 108:999–1007. 10.1007/s00421-009-1272-6 19967395

[96] Hickson RC Davis JR. Partial Prevention of Glucocorticoid-Induced Muscle Atrophy by Endurance Training. Am J Physiol. (1981) 241:E226–E32. 10.1152/ajpendo.1981.241.3.E226 7282923

[97] Holten MK Zacho M Gaster M Juel C Wojtaszewski JFP Dela F. Strength training increases insulin-mediated glucose uptake, GLUT4 content, and insulin signaling in skeletal muscle in patients with type 2 diabetes. Diabetes. (2004) 53:294–305. 10.2337/diabetes.53.2.294 14747278

[98] Wester VL Lamberts SW van Rossum EF. Advances in the assessment of cortisol exposure and sensitivity. Curr Opin Endocrinol Diabetes Obes. (2014) 21:306–11. 10.1097/MED.0000000000000077 24983396

[99] Russell E Kirschbaum C Laudenslager ML Stalder T de Rijke Y van Rossum EFC. Toward standardization of hair cortisol measurement: results of the first international interlaboratory round robin. Ther Drug Monit. (2015) 37:71–5. 10.1097/FTD.0000000000000148 25387254

[100] Wester VL Staufenbiel SM Veldhorst MAB Visser JA Manenschijn L Koper JW. Long-term cortisol levels measured in scalp hair of obese patients. Obesity. (2014) 22:1956–8. 10.1002/oby.20795 24852462

[101] Jackson SE Kirschbaum C Steptoe A. Hair cortisol and adiposity in a population-based sample of 2,527 men and women aged 54 to 87 years. Obesity. (2017) 25:539–44. 10.1002/oby.21733 28229550 PMC5324577

[102] Sauve B Koren G Walsh G Tokmakejian S Van Uum SHM. Measurement of cortisol in human hair as a biomarker of systemic exposure. Clin Invest Med. (2007) 30:E183–E91. 10.25011/cim.v30i5.2894 17892760

[103] Cummings DE. Endocrine mechanisms mediating remission of diabetes after gastric bypass surgery. Int J Obes. (2009) 33(Suppl 1):S33–40. 10.1038/ijo.2009.15 19363506

[104] Rubino F Forgione A Cummings DE Vix M Gnuli D Mingrone G. The mechanism of diabetes control after gastrointestinal bypass surgery reveals a role of the proximal small intestine in the pathophysiology of type 2 diabetes. Ann Surg. (2006) 244:741–9. 10.1097/01.sla.0000224726.61448.1b 17060767 PMC1856597

[105] Woods CP Corrigan M Gathercole L Taylor A Hughes B Gaoatswe G. Tissue specific regulation of glucocorticoids in severe obesity and the response to significant weight loss following bariatric surgery (BARICORT). J Clin Endocr Metab. (2015) 100:1434–44. 10.1210/jc.2014-4120 25603461

[106] Simonyte K Olsson T Naslund I Angelhed JE Lonn L Mattsson C Weight loss after gastric bypass surgery in women is followed by a metabolically favorable decrease in 11 beta-hydroxysteroid dehydrogenase 1 expression in subcutaneous adipose tissue. J Clin Endocr Metab. (2010) 95:3527–31. 10.1210/jc.2009-2472 20410231

[107] Rask E Simonyte K Lonn L Axelson M. Cortisol metabolism after weight loss: associations with 11 beta-HSD type 1 and markers of obesity in women. Clin Endocrinol. (2013) 78:700–5. 10.1111/j.1365-2265.2012.04333.x 22233384

[108] Methlie P Dankel S Myhra T Christensen B Gjerde J Fadnes D. Changes in adipose glucocorticoid metabolism before and after bariatric surgery assessed by direct hormone measurements. Obesity. (2013) 21:2495–503. 10.1002/oby.20449 23512832

[109] Diamant S Shafrir E. Modulation of activity of insulin-dependent enzymes of lipogenesis by glucocorticoids. Eur J Biochem. (1975) 53:541–6. 10.1111/j.1432-1033.1975.tb04097.x 237762

